# A Note on Blood Changes in Gas Poisoning

**Published:** 1918

**Authors:** 


***A Note on Blood Changes in Gas Poisoning***. By JAMES MILLER, Capt., R. A M. C. (T.) and HARRY FAINY, Capt., R.A.M.C. (T.). Abs. from the *Lancet*, Jan. 6, 1917.

The analysis of 14 cases leads the author to the following conclusions:

A gassing sufficient to produce symptoms lasting for some time gives rise to a more or less marked lymphocytosis.

This result is brought about only after a considerable lapse of time, probably 2 to 4 months at least. When the condition is once established it continues for about a year if symptoms of any kind persist.

There is apparently no marked change in the number of leucocytes per cubic millimeter. If anything, there is a slight reduction.

The author does not know whether the change is common to all types of gas poisoning or whether it is peculiar to one or more varieties.

